# Molecular Engineering of Ionization-Responsive Self-Assembled Hole-Transport Materials for High-Performance Inverted Perovskite Solar Cells

**DOI:** 10.1021/acs.orglett.6c02798

**Published:** 2026-08-07

**Authors:** Yogesh S. Tingare, Chaochin Su, Xin-Yi Chen, Yu-Chi Chang, Sheng-Hung Teng, Ja-Hon Lin, Chi-Wei Cheng, Yun-Qiao Wang, Kuan-Chieh Lu, Huang-Yueh Liu, Wen-Ren Li

**Affiliations:** † Institute of Organic and Polymeric Materials, 34877 National Taipei University of Technology, Taipei 106344, Taiwan; ‡ Department of Chemistry, 34911 National Central University, Zhongli 32001, Taiwan; § Department of Electro-Optical Engineering, National Taipei University of Technology, Taipei 106344, Taiwan

## Abstract

Self-assembled hole-transporting materials (HTMs) offer considerable potential for perovskite solar cells (PSCs). However, interfacial optimization is critical for high performance. Twisted donor–acceptor–donor HTMs incorporating double anchoring groups were developed to improve energy alignment and defect passivation. Ionization was observed to enhance interfacial interactions and wettability, facilitate nucleation, and suppress recombination, resulting in a 20.28% efficiency for ionic HTM **XYC108**. These findings highlight the potential of ionized SAM-based HTMs for efficient inverted PSCs.

## Graphical abstract

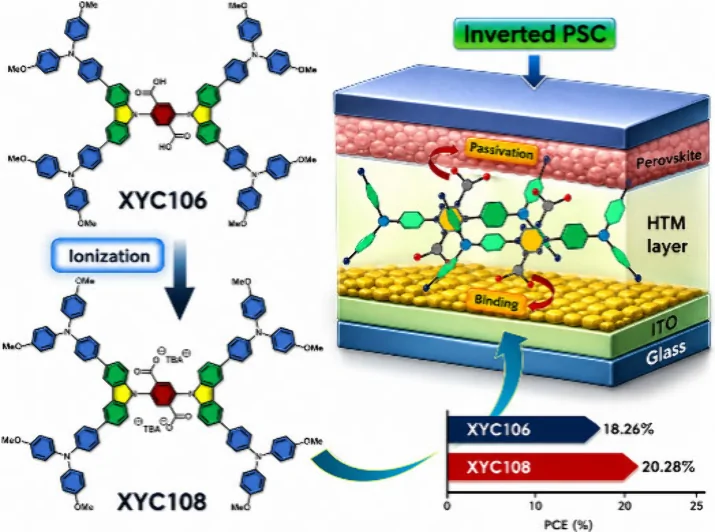

Metal halide perovskite solar cells (PSCs) have become prominent in photovoltaic research due to their high power conversion efficiencies (PCEs) and cost-effective fabrication methods.^1–6^ Since their initial demonstration in 2009, certified efficiencies have exceeded 27%, primarily attributed to the superior optoelectronic properties of perovskite absorbers.^7,8^ Among the essential functional layers, hole-transporting materials (HTMs) are critical for facilitating efficient hole extraction, minimizing charge recombination, and improving device stability.^9–24^ Therefore, advancing efficient and stable HTMs is vital to further enhancing PSC performance.^25^

Self-assembled materials (SAMs) have emerged as promising HTMs for PSCs due to their ability to form highly ordered, compact molecular layers with precise control over thickness and interfacial properties.^26–31^ The tunable molecular structures of SAMs facilitate energy-level alignment, efficient interfacial charge transfer, surface defect passivation, and improved device stability.^26,27,32,33^ These characteristics contribute to reduced nonradiative recombination and enhanced photovoltaic performance.^34–37^ SAM-based HTMs offer additional advantages, including reduced material consumption, simplified processing, and the potential for low-cost, large-scale fabrication.^38^ Typically, SAMs are composed of a π-conjugated terminal group for hole extraction and transport, a linker, and an anchoring unit that binds to the transparent conductive oxide substrate.^26,39^ Common terminal groups include carbazole, phenothiazine, acridine, and triphenylamine.^40^ Carbazole is particularly notable for its facile functionalization, excellent charge-transport properties, chemical stability, and tunable optoelectronic characteristics.^41^ Linkers may be conjugated, such as benzene, thiophene, and benzothiazole, or nonconjugated alkyl chains. Anchoring groups, including phosphonic, carboxylic, and boric acids, ensure strong substrate attachment and a dense monolayer.^26,39,40,42–44^

Structurally, SAM-based HTMs are analogous to the dyes employed in dye-sensitized solar cells (DSSCs), allowing for the transfer of established DSSC design strategies.^3,45–50^ For instance, converting the N3 dye into its salt form, N719, enhances solubility, increases cell voltage, and improves stability due to stronger electrostatic interactions with the titanium dioxide (TiO_2_) surface, establishing N719 as a benchmark DSSC dye.^51–54^ Dye aggregation, a significant challenge in DSSCs, results from densely packed molecules on TiO_2_, which disrupts monolayer formation and diminishes device performance.^54–56^ A comparable issue is observed in PSCs, where nonuniform SAM coverage reduces efficiency.^57,58^ In DSSCs, coadsorbents are effective in suppressing dye aggregation,^59–61^ whereas in PSCs, strategies such as coadsorbing 3-mercaptopropionic acid with 2PACz, employing methyl-substituted acridine cores, and introducing twisted molecular structures mitigate SAM aggregation and enhance film uniformity.^58,62–64^ Implementing these approaches in SAM HTM design is likely to further improve PSC efficiency and stability.

Two novel small-molecule HTMs, **XYC106** and **XYC108**, were developed for PSCs (Figure 1a). Drawing on the ionic modification strategy employed in DSSCs, where N3-to-N719 conversion enhances solubility, reduces aggregation, and strengthens interfacial interactions, ionic functionality was integrated into the self-assembled HTM design. **XYC108** was synthesized by converting the acid groups of **XYC106** into ionic salts, resulting in stronger electrostatic interactions, improved molecular organization, and more efficient charge transport. Both carbazole-based HTMs feature a donor–acceptor–donor (D–A–D) architecture, in contrast to the conventional donor−π–acceptor (D-π-A) design. This configuration facilitates parallel alignment with the perovskite surface and promotes efficient charge transport. The acid or salt groups serve dual roles as indium tin oxide (ITO) anchoring sites and as agents for perovskite defect passivation. The twisted, noncoplanar molecular structure further inhibits aggregation. As a result, **XYC108** achieved a PCE of 20.28%, outperforming both **XYC106** (18.26%) and poly­(3,4-ethylenedioxythiophene):polystyrenesulfonate (**PEDOT:PSS**) (14.74%). These findings establish ionic engineering as an effective strategy for the development of advanced SAM-based HTMs.

**Figure 1:**
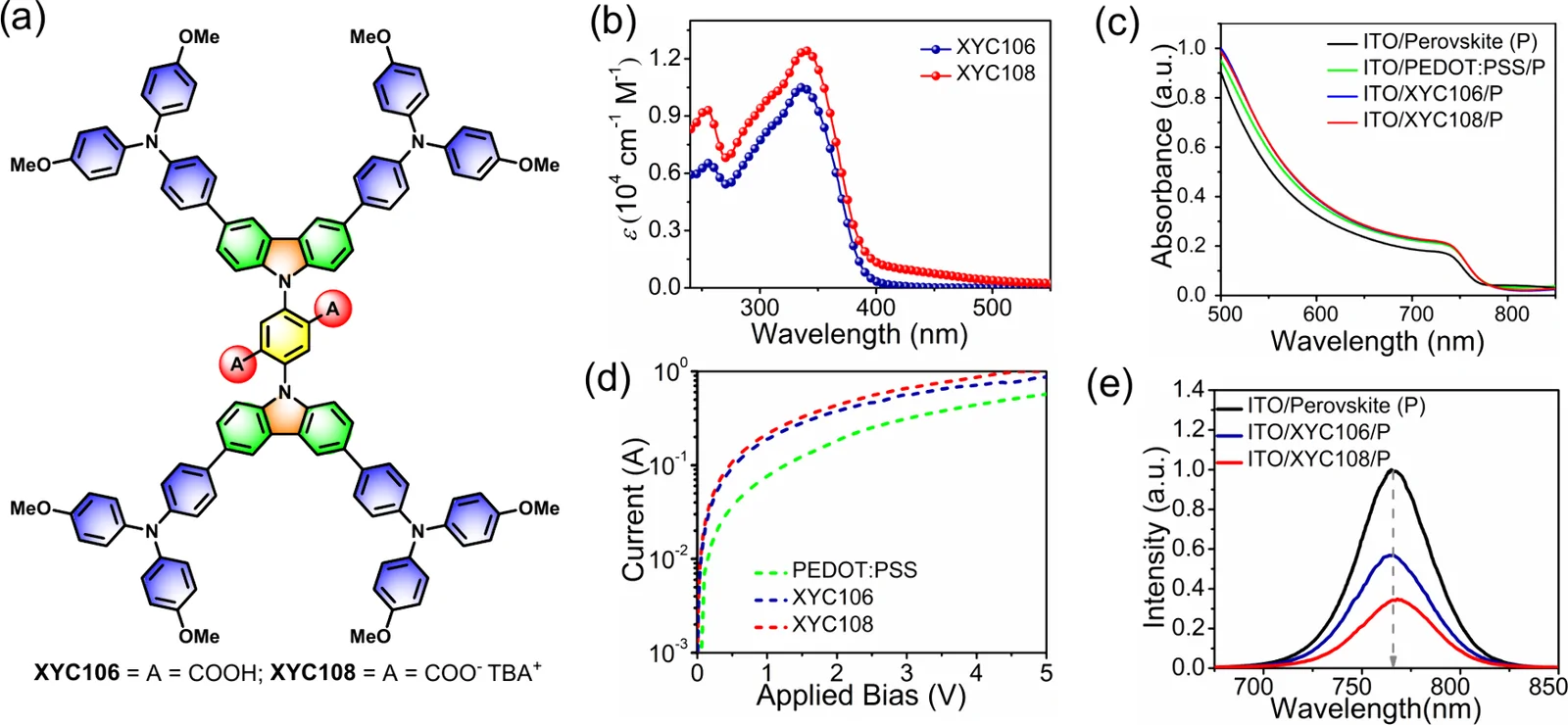
(a) Molecular structure of SAM HTMs **XYC106** and **XYC108**. (b) UV–vis absorption spectra of **XYC106** and **XYC108** (c) Absorption spectra of perovskite films deposited atop various HTM layers. (d) SCLC measurement of HTM mobility with the ITO/HTM/Au configuration. (e) Steady-state photoluminescence results for perovskites and the perovskite/HTM interface.

Scheme 1 presents the synthetic routes for HTMs **XYC106** and **XYC108**; detailed procedures are provided in the Supporting Information (SI). The chemical structures of these compounds were confirmed by ^1^H and ^13^C nuclear magnetic resonance spectroscopy and mass spectrometry. Ultraviolet–visible (UV–vis) spectroscopy was employed to evaluate their photophysical properties in a dichloromethane solution. As shown in Figure 1b and Table 1, **XYC106** and **XYC108** display similar absorption spectra, with maxima at 338 and 340 nm, respectively, which are attributed to the π–π* transitions of the triphenylamine (TPA)-functionalized carbazole core. This observation reflects their comparable conjugated structures. In inverted PSCs, high HTM transparency is critical for minimizing parasitic absorption and maximizing photon harvesting. The UV–vis absorption and transmission spectra of **XYC106** and **XYC108** thin films (Figure S1) demonstrate excellent transparency, with transmittance exceeding 95% across the range of 400–800 nm, thereby enabling efficient light penetration into the perovskite layer. UV–vis spectra of perovskite films deposited on ITO/HTM substrates (Figure 1c) reveal similar absorption profiles in the range of 500–800 nm. However, the absorbance decreases in the following order: **XYC108** > **XYC106** > **PEDOT:PSS**. This indicates a modest improvement in light harvesting for perovskite films on **XYC108** and suggests a more optically favorable interface for inverted PSCs.

**Scheme 1:**
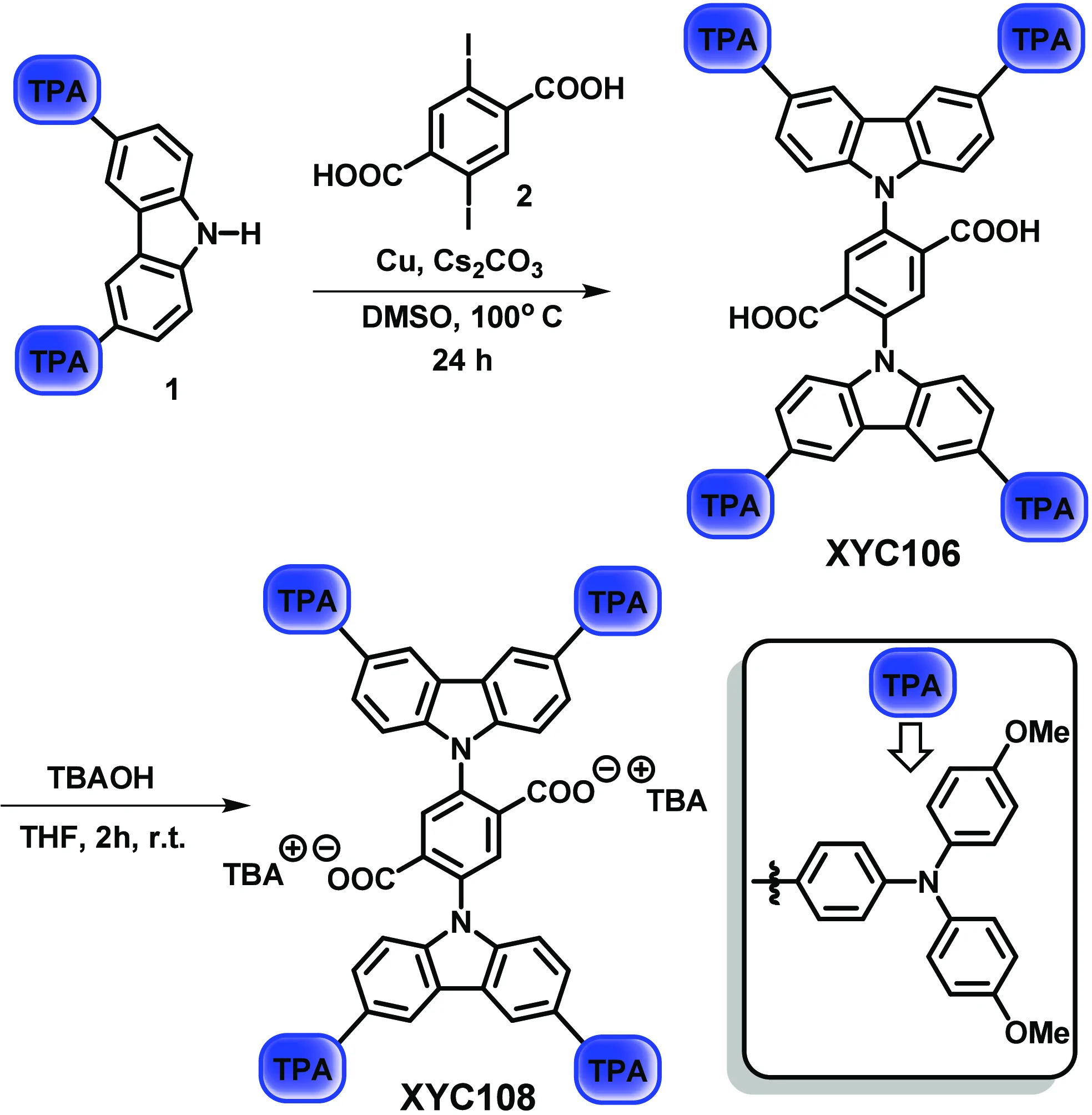
Synthesis of HTMs **XYC106** and **XYC108**

**Table 1:** Optical and Electrochemical Properties of HTMs **XYC106** and **XYC108**

	λ_max_ ^a^ (nm)				
HTM	solution^a^	film^b^	*E*_g_^opt^ (eV)	*E*_HOMO_ ^c^ (eV)	*E*_LUMO_ ^d^ (eV)
**XYC106**	253, 338	355	3.08	–5.05	–1.97
**XYC108**	254, 340	365	3.16	–5.23	–2.07

a Absorption spectra were measured in dichloromethane solutions.

b Absorption spectra of films.

c The optical bandgap was calculated using the formula 1240/λ onset.

d *E*_LUMO_ = *E*_HOMO_ + *E*_g_^opt^.

The electrochemical properties of self-assembly-based HTMs **XYC106** and **XYC108** were evaluated using cyclic voltammetry in tetrahydrofuran with ferrocene as an internal standard (Figure S2). UV–vis spectra revealed λ_onset_ values of 403 nm for **XYC106** and 392 nm for **XYC108**, corresponding to optical bandgaps (*E*_g_^opt^) of 3.08 and 3.16 eV, respectively. Oxidation potentials yielded highest occupied molecular orbital (HOMO) levels of −5.05 eV for **XYC106** and −5.23 eV for **XYC108** and lowest unoccupied molecular orbital (LUMO) levels of −1.97 and −2.07 eV, respectively. The deeper HOMO level and wider bandgap of **XYC108** indicate enhanced energy-level alignment and potentially more efficient hole extraction in inverted PSCs.

Hole-transport properties were assessed using space-charge-limited current (SCLC) measurements with an ITO/HTM/Au device configuration (Figure 1d). **XYC106** and **XYC108** exhibited hole mobilities of 3.96 × 10^–4^ and 5.32 × 10^–4^ cm^2^ V^–1^ s^–1^, respectively, both surpassing that of **PEDOT:PSS** (2.59 × 10^–4^ cm^2^ V^–1^ s^–1^). The superior mobility of **XYC108** suggests enhanced intermolecular coupling and improved interfacial ordering, which facilitate efficient hole transport. Photoluminescence (PL) and time-resolved photoluminescence (TRPL) measurements demonstrated improved hole extraction in HTM-based films, with **XYC108** showing the most pronounced PL quenching (Figure 1e). This result indicates reduced recombination and a lower density of interfacial traps. TRPL analysis (Figure S3) further revealed the fastest interfacial charge transfer for **XYC108** (τ_1_: **XYC108** < **XYC106** < **PEDOT:PSS** < pristine perovskite), confirming accelerated hole transfer and effective defect passivation. Collectively, these enhanced transport and extraction characteristics of **XYC108** reduce recombination losses and thereby improve device performance.

Effective suppression of interfacial charge recombination requires precise control of perovskite crystallization through the underlying HTM. Scanning electron microscopy (Figure 2a,b) demonstrates that **XYC108** yields larger and more uniform grains with fewer grain boundaries compared to **XYC106** and **PEDOT:PSS** (Figure S4), suggesting reduced trap density and improved charge transport. This enhancement is attributed to the optimized wettability (insets of panels a and b of Figure 2 and Figure S4) of **XYC108**, whose moderate hydrophobicity (57.67°) facilitates controlled nucleation and compact film formation, in contrast to **XYC106** (47.54°) and **PEDOT:PSS** (13.26°). Atomic force microscopy (Figure S5) further indicates that perovskite films on **XYC106** (8.15 nm) and **XYC108** (8.04 nm) are smoother than those on **PEDOT:PSS** (18.16 nm). Additionally, X-ray diffraction (Figure 2c) reveals sharper diffraction peaks, confirming enhanced crystallinity, reduced nonradiative recombination, and improved charge collection, all of which contribute to superior PSC performance.

**Figure 2:**
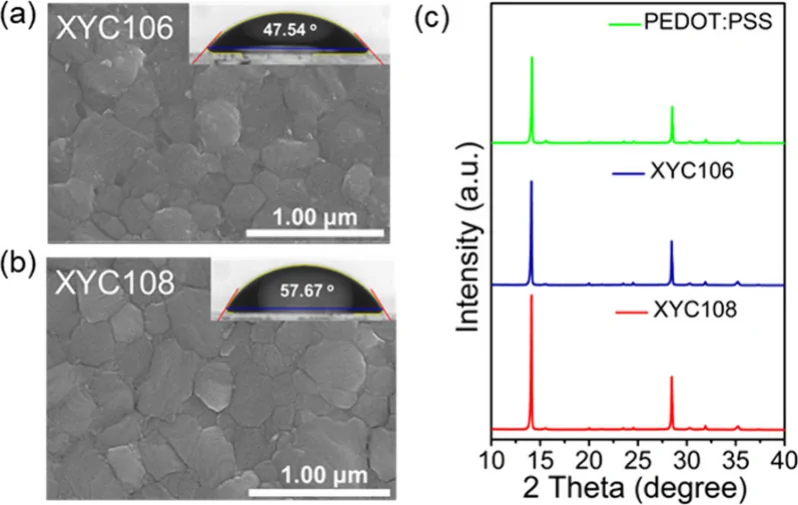
SEM images of top views of (a) **XYC106** and (b) **XYC108** of perovskite devices. The insets show the contact angles of the HTMs. (c) XRD of perovskite films atop **PEDOT:PSS**, **XYC106**, and **XYC108**.

PSCs with an inverted architecture (ITO/HTM/MAPb­(I_0.9_Cl_0.1_)_3_/PCBM/BCP/Ag) were fabricated using **XYC106**, **XYC108**, and **PEDOT:PSS** as HTMs (Figure 3a). The current density–voltage (*J*–*V*) curves with forward and reverse scans and photovoltaic parameters are presented in Figure 3b, Figure S6a, and Table 2. Devices employing **XYC106** with a forward scan direction achieved a PCE of 18.26%, an open-circuit voltage (*V*_OC_) of 1.03 V, a short-circuit current density (*J*_SC_) of 22.34 mA cm^–2^, and a fill factor (FF) of 80.12%, surpassing that of **PEDOT:PSS** (14.74%) due to reduced interfacial recombination facilitated by its D–A–D structure and acid-induced defect passivation. Conversion of acid groups into ionic salts yielded **XYC108**, which further increased the PCE to 20.28% (*V*_OC_ = 1.11 V, *J*_SC_ = 22.92 mA cm^–2^, and FF = 80.19%). This improvement is ascribed to enhanced energy-level alignment, diminished trap-assisted recombination, improved perovskite crystallinity, and more effective defect passivation. X-ray photoelectron spectroscopy analysis (Figure 3c) indicated a positive shift in the Pb 4f binding energies for **XYC106**- and **XYC108**-based films, confirming strong interfacial interactions and effective passivation of undercoordinated Pb^2+^ defects by the carbonyl group. Collectively, these synergistic effects suppress nonradiative recombination, improve film quality, and enhance the efficiency and stability of inverted PSCs.

**Table 2:** Photovoltaic Parameters of PSCs Using **PEDOT:PSS**, **XYC106**, and **XYC108**

HTM	*V*_OC_ (V)	*J*_SC_ (mA/cm^2^)	FF (%)	*Η* (%)
**PEDOT:PSS**	0.98	18.56	80.79	14.74
**XYC106**	1.03	22.34	80.12	18.26
**XYC108**	1.11	22.92	80.19	20.28

**Figure 3:**
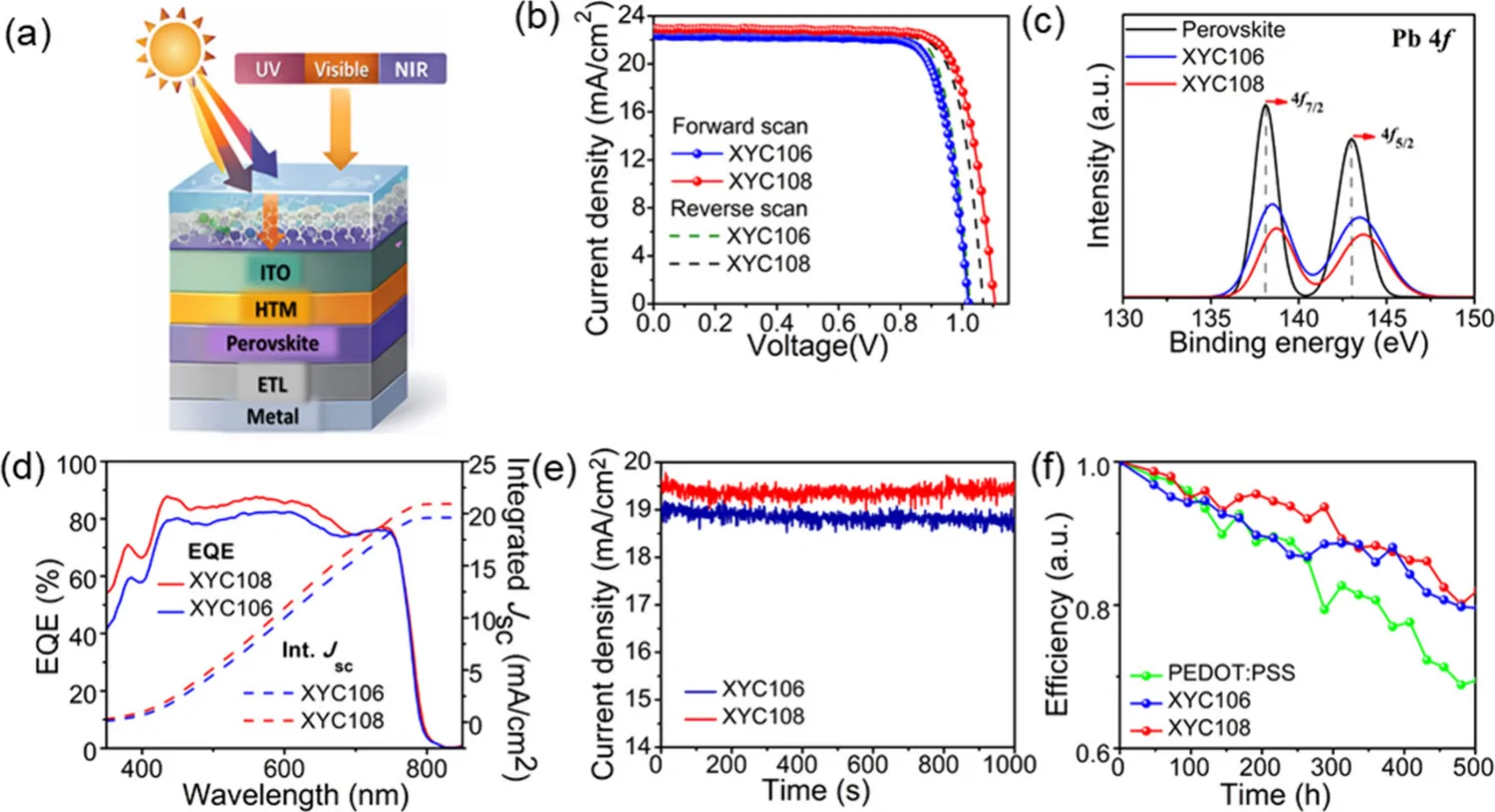
(a) Schematic representation of the geometry of an inverted PSC device. (b) *J*–*V* characteristic curves with forward and reverse scans for devices employing various HTMs. (c) XPS analysis of the Pb 4f peaks of the ITO/HTM/perovskite structure. (d) EQE spectra with integrated *J*_SC_ values of the PSCs with HTMs. (e) Steady-state photocurrent outputs of PSCs with different HTMs. (f) Long-term stability testing of the PSCs incorporated with HTMs.

Figure 3d and Figure S6b present broad external quantum efficiency (EQE) responses from 300 to 800 nm for all devices. The EQE of **XYC108** is higher than that of **XYC106**, indicating more efficient hole extraction, with integrated current densities of 20.93 and 19.62 mA cm^–2^, respectively. Reproducibility was assessed using 15 devices (Figure S7), resulting in average PCEs of 17.19% for **XYC108** and 15.76% for **XYC106**, both surpassing that of **PEDOT:PSS** (13.46%). As illustrated in Figure 3e, devices based on **XYC106** and **XYC108** achieved stabilized *J*_SC_ values of 21.82 and 22.60 mA cm^–2^, respectively, with negligible decay over 1000 s. Stability assessments (Figure 3f) indicate enhanced durability, with **XYC106** and **XYC108** retaining 79% and 81% of their initial PCEs, respectively, after 500 h compared to less than 69% for **PEDOT:PSS**.

In summary, ionization is shown to be an effective strategy for enhancing self-assembled HTMs. The twisted D–A–D ionic HTM design facilitates optimized energy alignment, strong substrate binding, and defect passivation via dual anchoring groups. Incorporation of ionic functionalities strengthens electrostatic interactions, suppresses charge recombination, and promotes uniform perovskite crystallization. Relative to **XYC106**, **XYC108** exhibits superior hole mobility and reduced interfacial resistance, resulting in a PCE of 20.28% due to an increased current output and minimized losses. These results establish ionized self-assembly-based HTMs as versatile and scalable platforms for high-performance inverted PSCs.

## Supplementary Material



## Data Availability

The data underlying this study are available in the published article and its Supporting Information.

## Notes

Synthetic procedures and details of the preparation and characterization of PSC devices, UV–vis transmittance spectra, cyclic voltammograms, time-resolved photoluminescence spectra, SEM image, AFM images, contact angle *J*–*V* characteristic curve, and EQE spectra of **PEDOT:PSS**, histograms of the PCE distribution of HTMs, fitting details of the biexponential function in TRPL of glass/perovskite substrates based on different HTMs, and ^1^H and ^13^C NMR spectra (PDF)
