# Supplementary material for: Molecular Engineering of Ionization-Responsive Self-Assembled Hole-Transport Materials for High-Performance Inverted Perovskite Solar Cells
Source: Org Lett. 2026 Aug 7;28(33):10495–500. doi: 10.1021/acs.orglett.6c02798 (PMC13505290; doi:10.1021/acs.orglett.6c02798)
Supplement: Supplementary file 1 [file ol6c02798_si_001.pdf]

Supporting Information

Molecular Engineering of Ionization-Responsive Self-Assembled Hole-Transport Materials for High-Performance Inverted Perovskite Solar Cells

Yogesh S. Tingare[a,*], Chaochin Su[a,*], Xin-Yi Chen[b], Yu-Chi Chang[a], Sheng-Hung Teng[b], Ja-Hon Lin[c], Chi-Wei Cheng[b], Yun-Qiao Wang[a], Kuan-Chieh Lu[b], Huang-Yueh Liu [a], Wen-Ren Li [b,*]

[a]Prof. Y. S. Tingare, Prof. C. Su,

Institute of Organic and Polymeric Materials, National Taipei University of Technology, Taipei 106344, Taiwan

E-mail: f12098@ntut.edu.tw; f10913@mail.ntut.edu.tw

[b]Prof. W.-R. Li

Department of Chemistry, National Central University, Zhongli 32001, Taiwan

E-mail: ch01@ncu.edu.tw

[c]Prof. J.-H. Lin.

Department of Electro-Optical Engineering, National Taipei University of Technology, Taipei 106344, Taiwan

1. Materials: Silica gel (Scharlau 60, 230-240 mesh) was used for flash chromatography. Reaction progress was monitored by thin-layer chromatography (TLC) on aluminum-coated Merck Kieselgel 60 F254 plates, developed with iodine or ultraviolet light. All intermediates and final products were characterized by ^1H and ^{13}C nuclear magnetic resonance (NMR) spectroscopy using a Bruker Avance 500 MHz spectrometer. Chemical shifts (δ) and coupling constants (*J*) are reported in parts per million (ppm) and hertz (Hz), respectively. Multiplicities are indicated as s (singlet), d (doublet), t (triplet), q (quartet), p (pentet), m (multiplet), and b (broad). Matrix-assisted laser desorption ionization-time-of-flight (MALDI-TOF) mass spectra were obtained with a Bruker Daltonics flex Analysis spectrometer. Cyclic voltammetry (CV) was conducted using a CHI 627C electrochemical workstation with a glassy carbon working electrode, a silver wire ($Ag/AgNO_3$ in acetonitrile) reference electrode, a platinum wire counter electrode, and 0.1 M tetrabutylammonium hexafluorophosphate as the supporting electrolyte. Cyclic voltammograms of hole-transport materials (HTMs) were typically recorded at a scan rate of 50 mV s^{-1}. Ferrocene was used as an internal calibration standard. Thermogravimetric analysis (TGA) was performed on a TA Instruments system at a ramp rate of 10 ˚C/min under nitrogen from 100 to 600 ˚C. A Shimadzu UV-vis-NIR spectrophotometer was employed to assess the optical properties of the films. Scanning electron microscopy (SEM) and X-ray diffraction (XRD) analyses were carried out using a Bruker ECO D8 series instrument to examine the morphology of perovskite films. Time-resolved photoluminescence (PL) spectra were measured with a spectrofluorometer (FS5;

Edinburgh Instruments) using 405 nm excitation. Contact angle measurements were performed with a Phoenix 300 (Surface Electro-Optics Co., Ltd.) using de-ionized water as the solvent.

2. Synthesis procedures

Synthesis of XYC106: Compound **1** (0.195 g, 0.252 mmol, 2.1 eq.) and 2,5-diiodoterephthalic acid (0.050 g, 0.119 mmol, 1 eq.) were dissolved in 1.2 mL of DMSO. Cesium carbonate (0.155 g, 0.476 mmol, 4 eq.) and copper powder (0.002 g, 0.024 mmol, 0.2 eq.) were then added, and the mixture was heated at 100˚C for 24 hours under an argon atmosphere. The reaction was quenched with 5% aqueous HCl to adjust the pH to 2. The mixture was extracted with dichloromethane and washed with brine. The organic layer was dried over anhydrous magnesium sulfate, filtered, and concentrated under reduced pressure. The resulting residue was purified by column chromatography (hexane:THF = 5:1 to 1:5), yielding **XYC106** (0.136 g, 66%) as a yellow solid. ^{1}H NMR (600 MHz, d-DMF) δ 13.82 (s, 2H), 8.74 (s, 4H), 8.48 (s, 2H), 7.83 (d, J = 8.2 Hz, 4H), 7.77 (d, J = 8.5 Hz, 8H), 7.55 (d, J = 8.5 Hz, 4H), 7.15 (d, J = 8.9 Hz, 16H), 7.02-7.00 (m, 24H), 3.84 (s, 24H). ^{13}C NMR (150 MHz, d-DMF) δ157.3, 148.8, 142.2, 141.9, 137.1, 134.6,134.3, 134.1, 128.5, 127.7,126.0, 125.5, 121.6, 119.3, 116.0, 111.2, 55.2. HRMS (MALDITOF, m/z) Calculated for $C_{112}H_{89}N_6O_{12}$ $[M+H]^+$ = 1709.6538, found 1709.6533.

Synthesis of XYC108: XYC106 (0.1 g, 0.058 mmol, 1 eq.) was dissolved in 0.58 mL of THF, followed by the dropwise addition of TBAOH (0.062 mL, 1.625 eq.). The reaction mixture was stirred for 2 hours under an argon atmosphere at room temperature. The resulting crude product was concentrated under vacuum and purified by recrystallization from acetone and hexane, affording **XYC108** (0.096 g, 76%) as a bisque solid. ^{1}H NMR (500 MHz, $CDCl_3$) δ 8.22 (d, J = 1.4 Hz, 4H), 7.97 (s, 2H), 7.71 (d, J = 8.6 Hz, 4H), 7.59 (dd, J = 1.5 and 8.6 Hz, 4H), 7.54 (d, J = 8.6 Hz, 8H), 7.10 (d, J = 8.95 Hz, 16H), 7.05 (d, J = 8.6 Hz, 8H), 6.85 (d, J = 9 Hz, 16H), 3.82 (s,

24H), 2.75 (s, 16H), 1.28 (s, 16H), 1.10 (q, J = 7 Hz, 16H), 0.78 (t, J = 7.2 Hz, 24H). ^{13}C NMR (75 MHz, CDCl3) δ 155.8, 147.3, 141.9, 141.3, 134.8, 133.5, 132.0, 130.6, 127.5, 126.3, 124.9, 123.6, 121.7, 117.0, 114.8, 112.5, 58.3, 55.6, 240.4, 19.6, 13.7. HRMS (MALDI-TOF, m/z) Calculated for $C_{112}H_{88}N_6O_{12}$ $[M-2TBA+2H]^-$ = 1708.6460, found 1708.6458,

3. Device fabrication and characterizations: Indium tin oxide (ITO) conducting glass substrates were cleaned in an ultrasonic cleaner for 15 minutes using detergent, deionized water, acetone, and isopropanol. Following cleaning, substrates were exposed to UV-ozone for 15 minutes and transferred to a nitrogen-filled glovebox. Within the glovebox, **XYC106** and **XYC108** (each at 2 mg/mL in anhydrous chlorobenzene) were prepared. Subsequently, 50 μL of each solution was spin-coated onto the ITO substrates at 3000 rpm for 30 seconds, followed by annealing at 110 ˚C for 30 minutes. The **PEDOT:PSS** stock solution was filtered through a 0.22 μm PVDF filter, spin-coated onto the ITO at 3500 rpm for 40 seconds, and heated at 110 ˚C for 30 minutes.

To prepare the mixed-halide perovskite ($MAPb(I_{0.9}Cl_{0.1})_3$) solution, MAI (206.65 mg), PbI2 (509.52 mg), and $PbCl_2$ (54.23 mg) were dissolved in 1000 μL of DMF:DMSO (885:1115) and stirred at 70 ˚C in a nitrogen-filled glovebox. The resulting perovskite solution (50 μL) was spin-coated onto the hole transport material (HTM) layers at 2700 rpm for 50 seconds. Thirty-five seconds before the end of spin-coating, 200 μL of toluene was applied to the perovskite layer. After antisolvent application, a 5 psi nitrogen gas stream was directed over the film for 30 seconds as part of the gas-assisted method. The films were annealed at 60 ˚C for 5 minutes, then at 100 ˚C

for 3 minutes. Once cooled, 50 μL of [6,6]-phenyl-C61-butyric acid methyl ester (PCBM, 20 mg/mL in chlorobenzene), filtered through a 0.45 μm PTFE filter, was spin-coated at 2000 rpm for 40 seconds and annealed at 90 ˚C for 5 minutes. After further cooling, 60 μL of bathocuproine (BCP, 0.5 mg/mL in isopropanol) was spin-coated at 4000 rpm for 50 seconds. A 100 nm-thick silver (Ag) electrode was then deposited by thermal evaporation in a vacuum deposition chamber at a pressure of 10^-7 torr.

4. Device characterizations: *J*-V characteristics were measured with Keithley 2400 source measurement units, operated at room temperature inside a glovebox. Photovoltaic performance was evaluated under 100 mW/cm² illumination using a calibrated solar simulator (Class 3A, SS-F5-ABA, Enli Technology). Light intensity was calibrated with a standard photovoltaic reference cell (SRC-2020 series, Enli Technology). The IVS-KA6000 software (Enli Technology) was used to control light intensity during measurements. The external quantum efficiency (EQE) spectrum was recorded using the QE-R Model (Enli Technology).

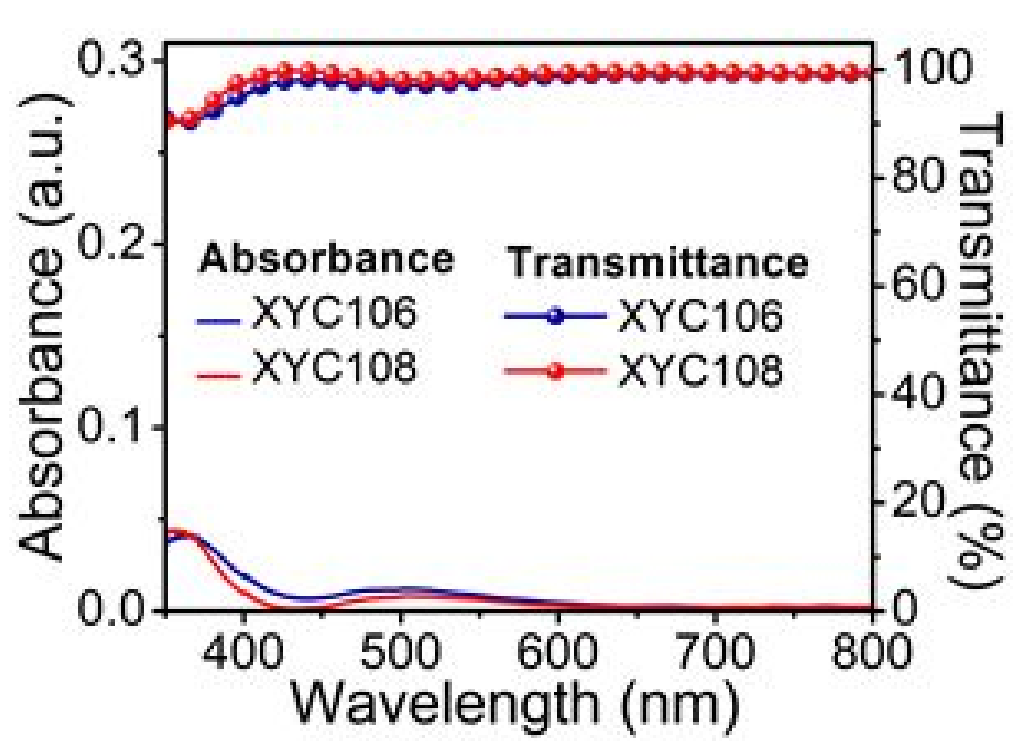

Figure S1. UV-vis transmittance spectra of **XYC106** and **XYC108** films.

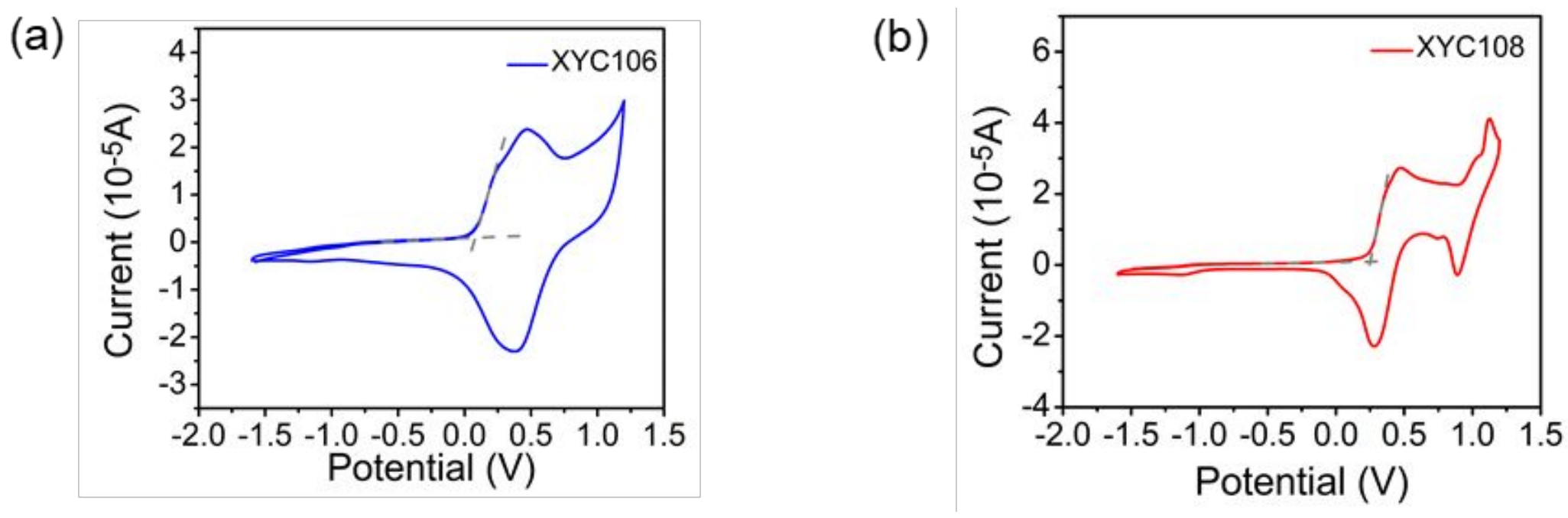

Figure S2. Cyclic voltammograms of (a) **XYC106** and (b) **XYC108**.

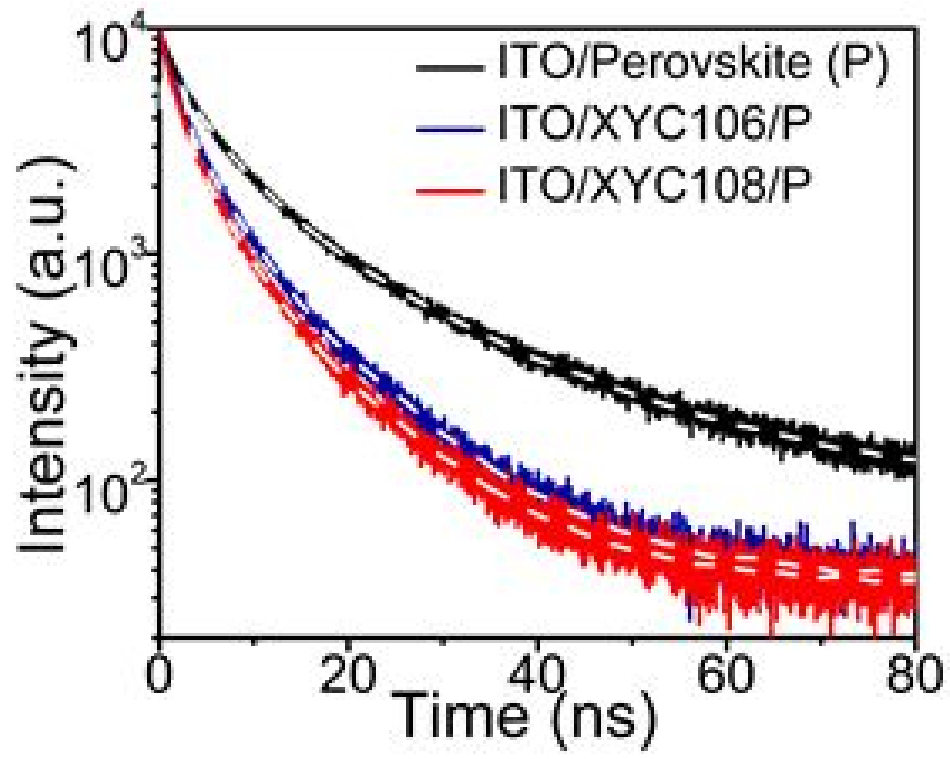

Figure S3. Time-resolved photoluminescence results for perovskites and the perovskite/HTM interface.

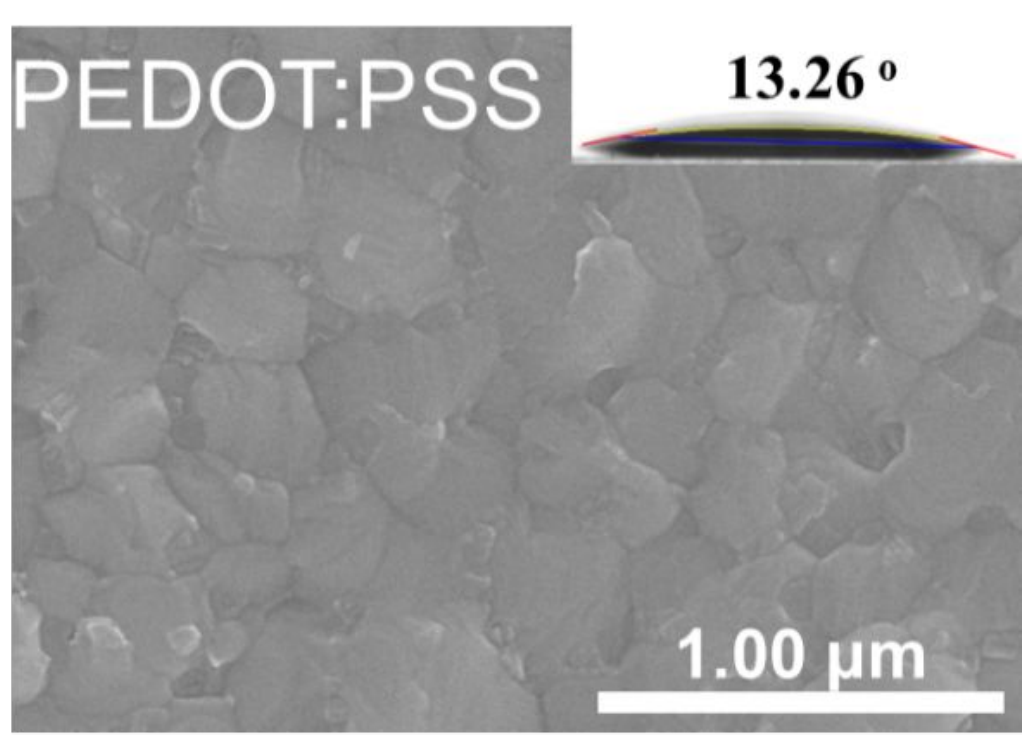

Figure S4. SEM image showing surface view of perovskite layer with **PEDOT:PSS** and inset contact angle measurements for **PEDOT:PSS**.

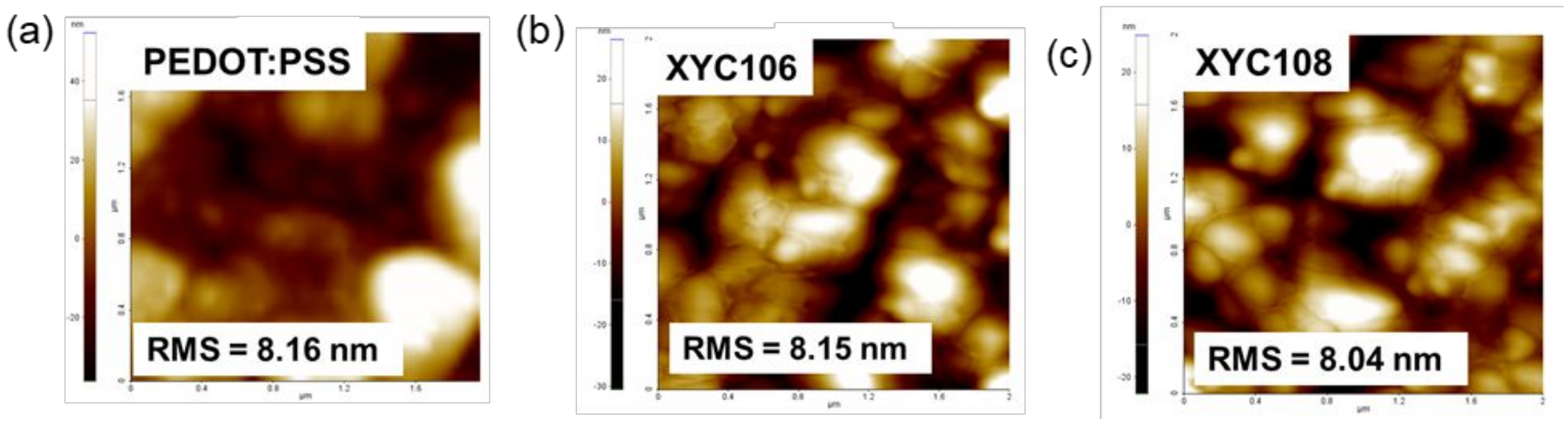

Figure S5. AFM images of (a) **PEDOT:PSS**, (b) **XYC106**, and (d) **XYC108** HTMs.

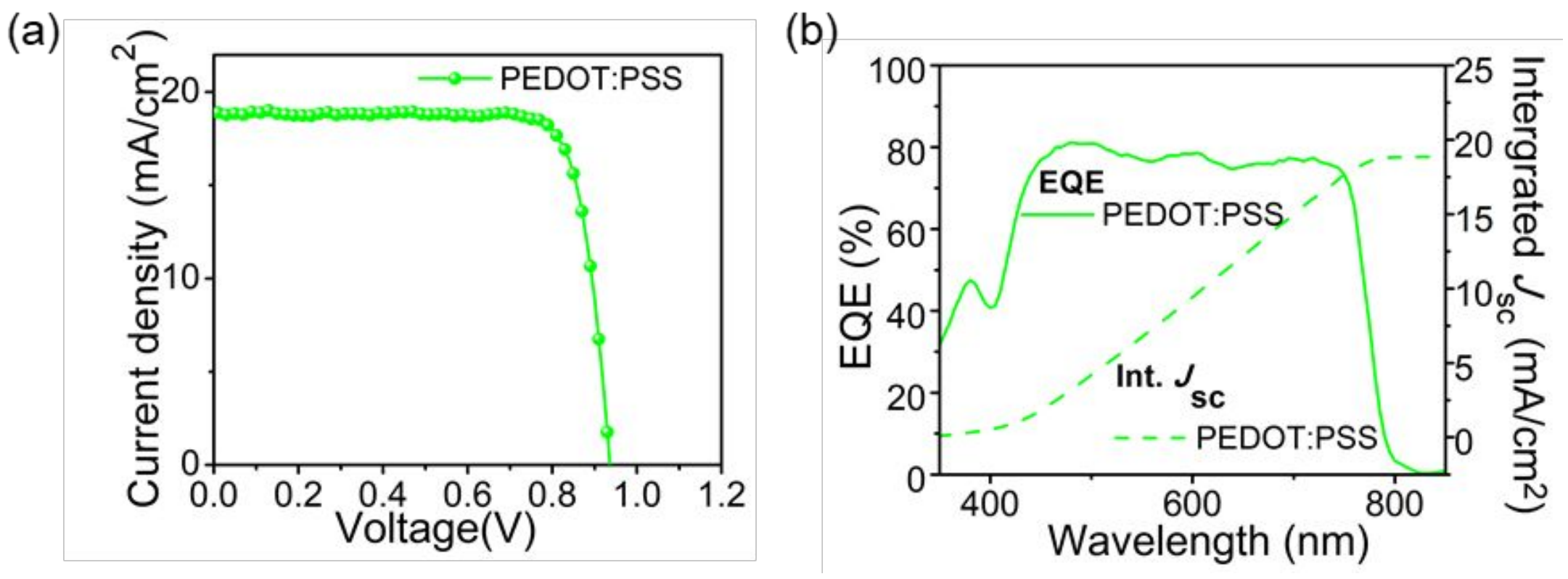

Figure S6. (a) *J*–V characteristic curve and (b) EQE spectra with integrated J_{SC} of the PSCs with **PEDOT:PSS**.

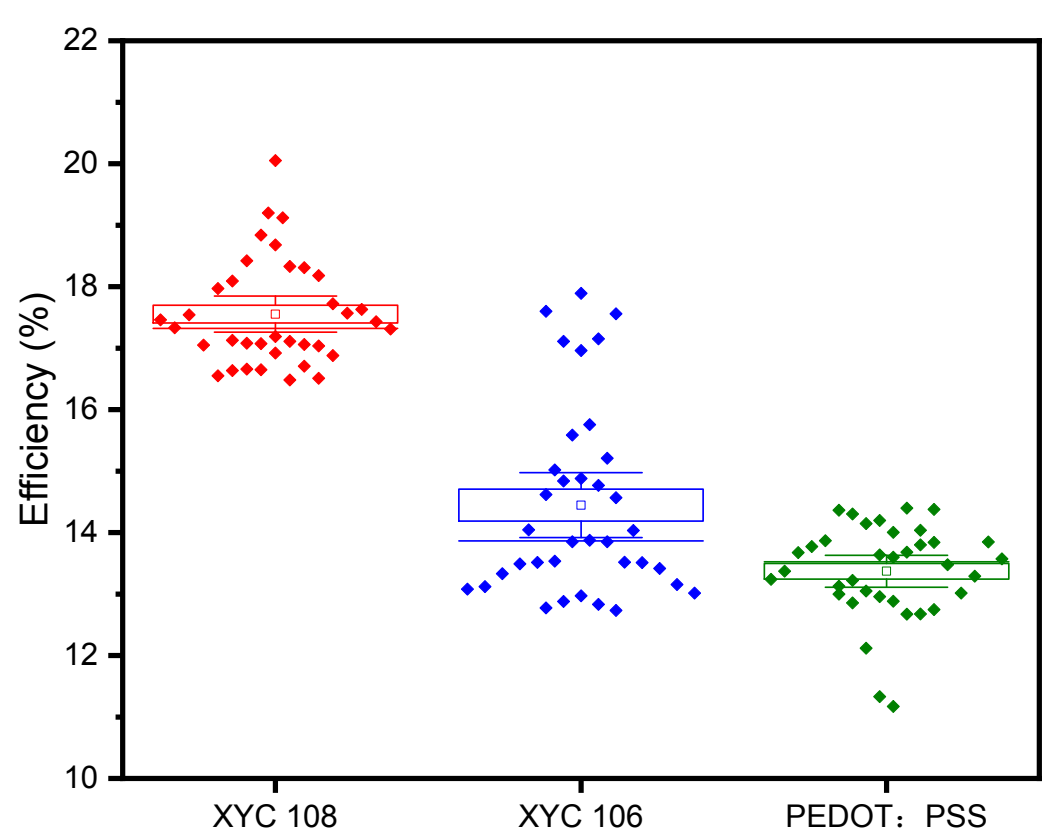

Figure S7. Histograms of the PCE distribution of HTMs **XYC108**, **XYC106** and **PEDOT:PSS**.

Table S1: Fitting Details of bi-exponential function in TRPL of glass/perovskite substrates based on different HTMs.

HTMs	A_1	τ_1	A_2	τ_2	τ_{Ave}
ITO/Perovskite	5283.4	2.6172	4224.0	12.330	6.9325
ITO/**PEDOT:PSS**/Perovskite	4510.2	2.3403	5163.5	8.9612	5.8743
ITO/**XYC106**/Perovskite	6037.1	1.8517	3918.7	7.7784	4.1845
ITO/**XYC108**/Perovskite	6065.0	1.6406	3763.0	6.9338	3.6673

^{1}H and ^{13}C spactra

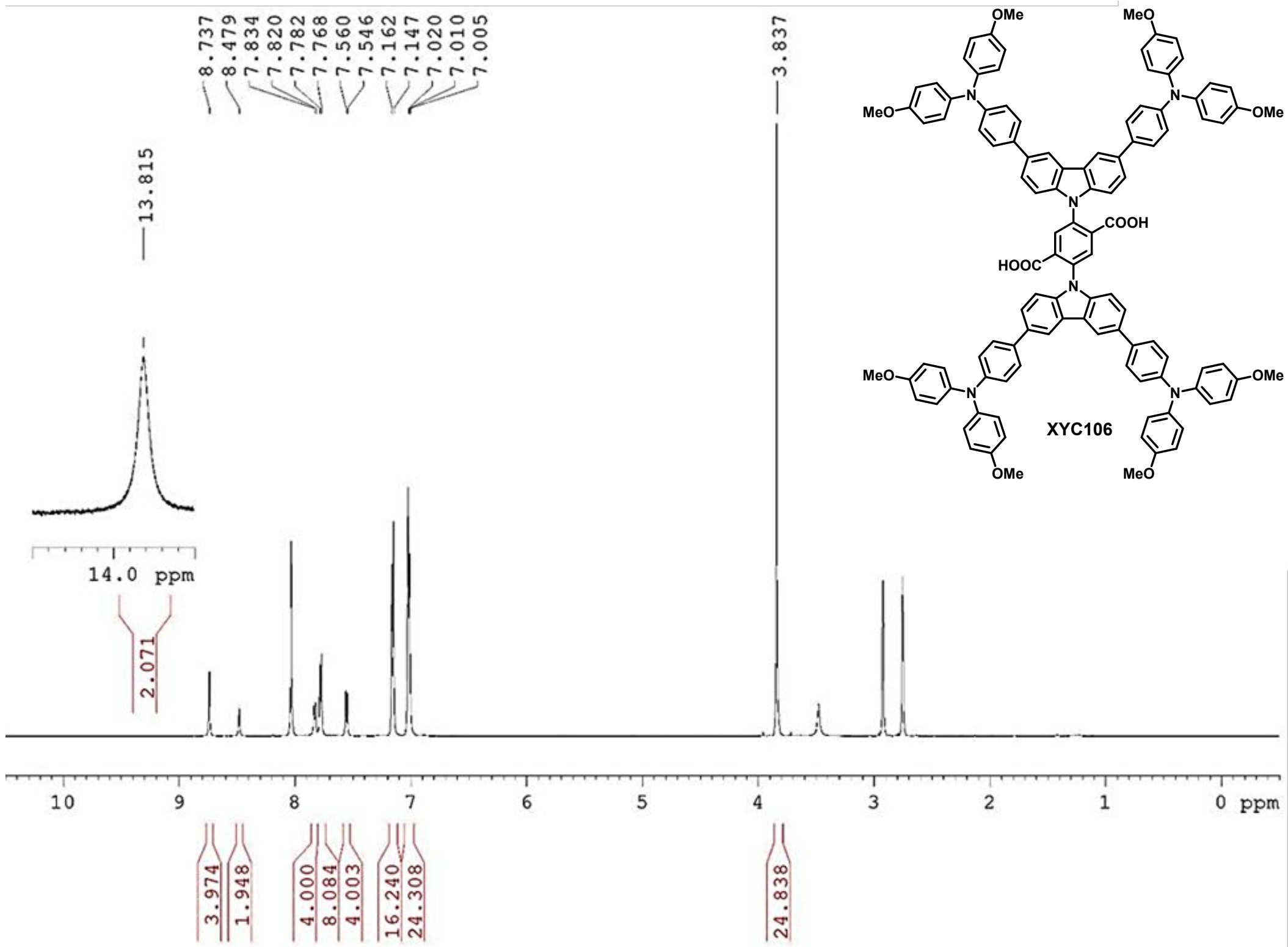

8.737
8.479
7.834
7.820
7.782
7.768
7.560
7.546
7.162
7.147
7.020
7.010
7.005
3.837
13.815
14.0 ppm
2.071
OMe
MeO
N
COOH
HOOC
XYC106
10 9 8 7 6 5 4 3 2 1 0 ppm
3.974
1.948
4.000
8.084
4.003
16.240
24.308
24.838

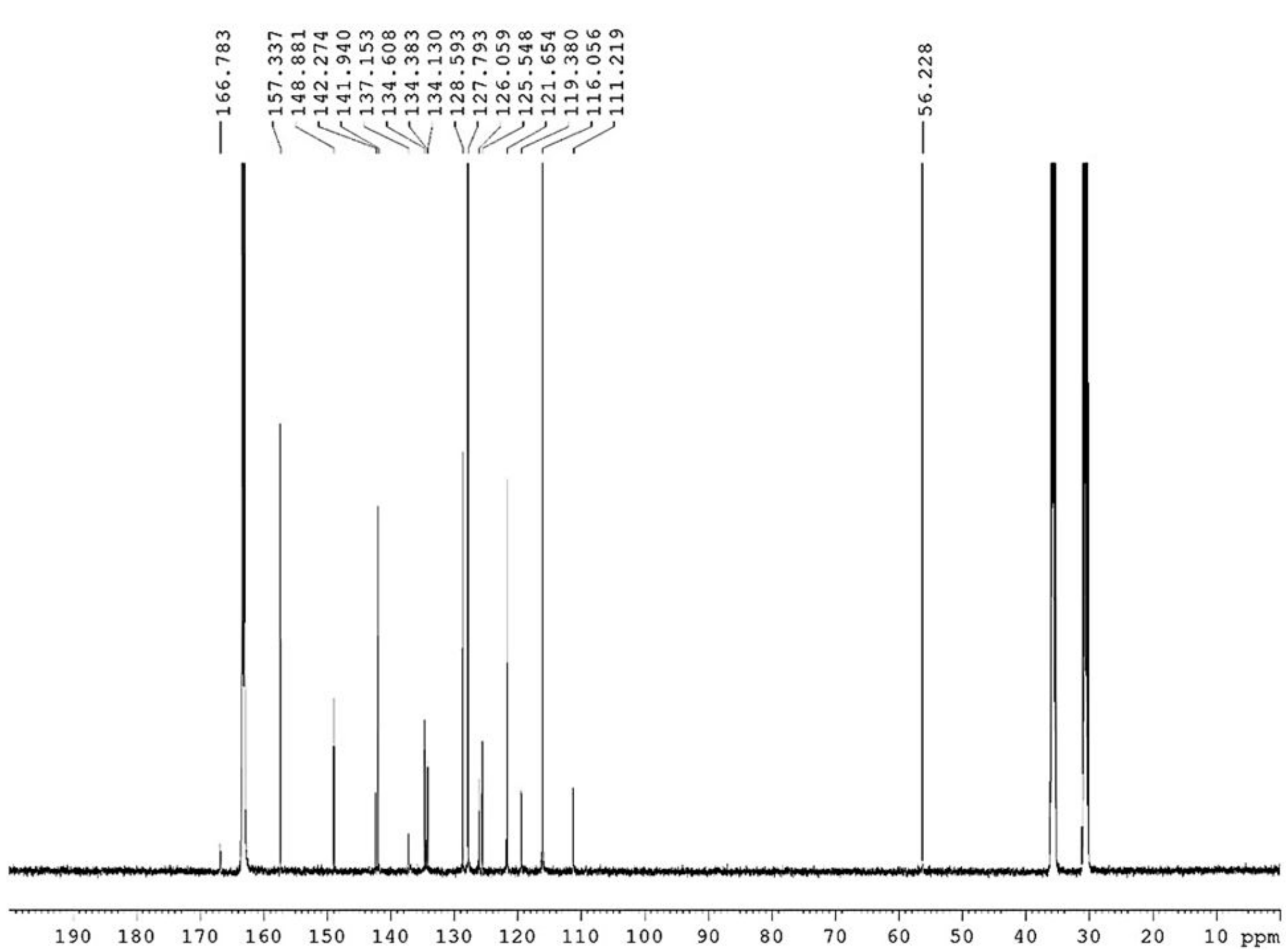

166.783
157.337
148.881
142.274
141.940
137.153
134.608
134.383
134.130
128.593
127.793
126.059
125.548
121.654
119.380
116.056
111.219
56.228
190 180 170 160 150 140 130 120 110 100 90 80 70 60 50 40 30 20 10 ppm

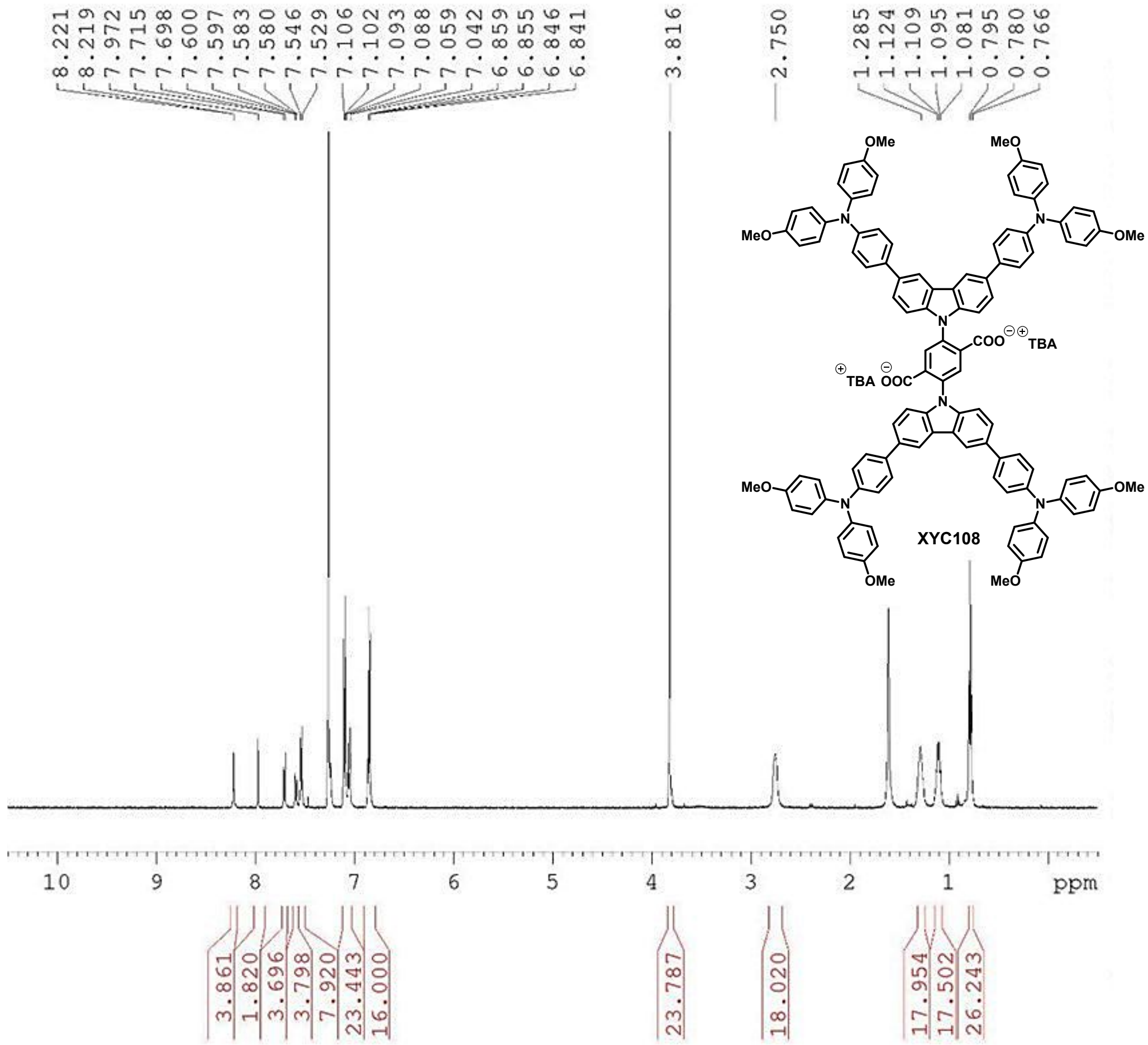
8.221
8.219
7.972
7.715
7.698
7.600
7.597
7.583
7.580
7.546
7.529
7.106
7.102
7.093
7.088
7.059
7.042
6.859
6.855
6.846
6.841
3.816
2.750
1.285
1.124
1.109
1.095
1.081
0.795
0.780
0.766
OMe
MeO
N
COO
TBA
OOC
XYC108
10
9
8
7
6
5
4
3
2
1
ppm
3.861
1.820
3.696
3.798
7.920
23.443
16.000
23.787
18.020
17.954
17.502
26.243

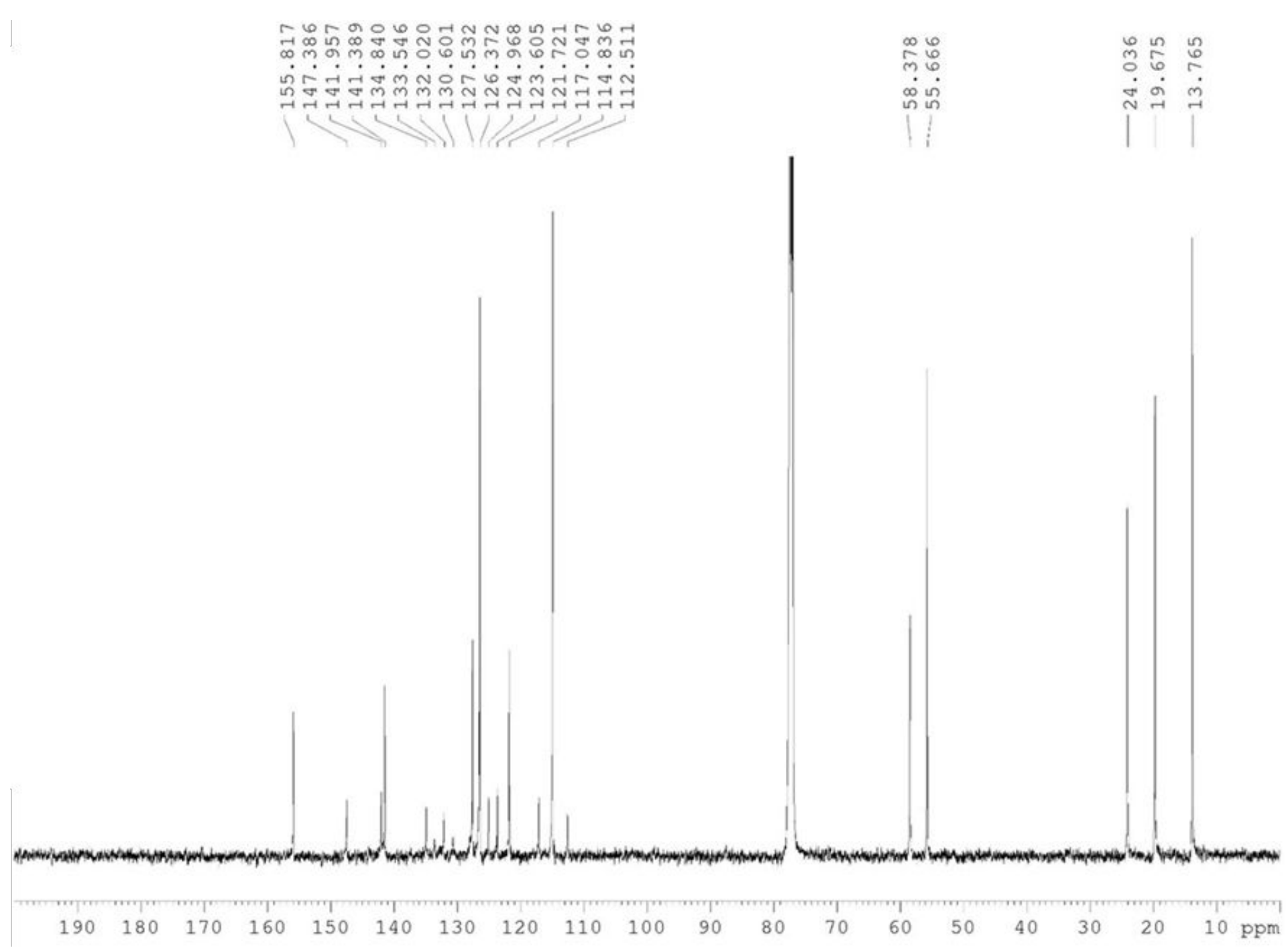
155.817
147.386
141.957
141.389
134.840
133.546
132.020
130.601
127.532
126.372
124.968
123.605
121.721
117.047
114.836
112.511
58.378
55.666
24.036
19.675
13.765
190
180
170
160
150
140
130
120
110
100
90
80
70
60
50
40
30
20
10 ppm
